# Learning Work Function via Implicit Reasoning on Electrostatic Potential Landscapes

**DOI:** 10.1002/advs.77166

**Published:** 2026-08-18

**Authors:** Haoyu Wan, Yue Wu, Tianhao Su, Deng Pan

**Affiliations:** ^1^ Materials Genome Institute Shanghai University Shanghai People's Republic of China; ^2^ Ministry of Education Key Laboratory of Silicate Cultural Relics Conservation Shanghai University Shanghai People's Republic of China

**Keywords:** 2D materials, contrastive learning, deep learning, electrostatic potential, work function

## Abstract

Precise work function engineering in two‐dimensional (2D) materials is pivotal for next‐generation nanoelectronic devices. However, current data‐driven approaches are often hampered by the scarcity of high‐precision data and a lack of physical interpretability. We propose a Graph‐Potential Cross‐Modal Contrastive Learning framework designed to uncover correlations between crystal and electronic structures. Rather than performing a direct scalar mapping, our approach respects the strict thermodynamic definition of the work function (Φ  = *E_vac_*  − *E_Fermi_*). By extracting the vacuum level from 1D PAEP morphology and predicting the Fermi level via an auxiliary head, the model accurately predicts work functions (*R* ^2^ =  0.902). This indicates an automatic extraction of features governing electron escape barriers. Additionally, the model demonstrates exceptional fidelity in morphological reconstruction; predicted skewness and kurtosis of the potential surface show near‐perfect linear correlation with DFT data (*R* ^2^ > 0.98), proving that it successfully decodes microscopic charge distribution details. This cross‐modal alignment paradigm drives artificial intelligence to transcend simple numerical fitting and learn physically informative representations, facilitating future potential‐contour‐based inverse material design.

## Graphical abstract

StructPot‐CLR establishes a cross‐modal contrastive learning framework that aligns the crystal structures of 2D materials with plane‐averaged electrostatic potential landscapes for physically informed work‐function prediction. The model achieves an MAE of 0.265 eV and an R^2^ of 0.902 on the held‐out test set while accurately preserving key morphological characteristics of the potential landscape. Attention analysis further reveals distinct attention patterns associated with surface‐passivating and bulk‐screened atoms, while the learned latent space supports potential‐profile‐guided retrieval and inverse screening of 2D materials.

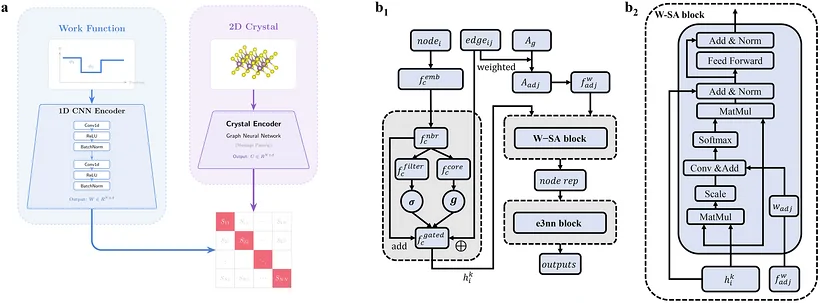

## 1 Introduction

The advent of two‐dimensional (2D) materials, epitomized by the isolation of graphene and the subsequent discovery of transition metal dichalcogenides (TMDs), has fundamentally altered the landscape of condensed matter physics and next‐generation electronics [1, 2, 3]. Due to their atomic thickness and reduced dielectric screening, these materials exhibit extraordinary sensitivity to surface conditions and external fields [4, 5, 6]. In the engineering of van der Waals (vdW) heterostructures and high‐performance field‐effect transistors (FETs), the surface work function (Φ) emerges as a paramount physical descriptor [7, 8, 9]. Defined thermodynamically as the energy required to remove an electron from the Fermi level (*E_F_*) to the vacuum level (*E_vac_*), Φ governs the band alignment at hetero‐interfaces and dictates the Schottky barrier height (SBH) at metal‐semiconductor contacts [10, 11]. The precise tuning of Φ is crucial for mitigating Fermi level pinning, optimizing ohmic contacts, and enhancing charge injection efficiency—factors that currently bottleneck the commercialization of 2D devices [12, 13, 14]. Furthermore, in the realm of electrocatalysis and corrosion protection, Φ serves as a direct indicator of the chemical potential; materials with higher Φ generally exhibit superior resistance to oxidative corrosion, a critical attribute for the longevity of atomic‐scale coatings [15, 16, 17]. Despite the critical role of work function engineering, establishing a comprehensive database of Φ for the rapidly expanding library of 2D materials remains an elusive goal. Experimental characterization techniques, such as Ultraviolet Photoelectron Spectroscopy (UPS) and Kelvin Probe Force Microscopy (KPFM), provide ground‐truth data but are inherently low‐throughput, expensive, and susceptible to environmental contamination [18, 19, 20]. Consequently, the field has heavily relied on first‐principles calculations based on Density Functional Theory (DFT). While DFT offers high accuracy, calculating surface properties is significantly more computationally demanding than bulk properties. It requires the construction of large supercells with vacuum layers to prevent spurious interactions between periodic images, leading to a computational scaling of *O*(*N* ^3^)  [21, 22]. This computational cost becomes prohibitive when screening the vast combinatorial space of potential 2D alloys, functionalized derivatives, and defective structures, thereby severely impeding data‐driven materials discovery [23, 24]. The emergence of materials informatics has introduced Machine Learning (ML) as a viable accelerator for property prediction. Early efforts utilizing classic descriptors‐such as Coulomb matrices or elemental fractions–‐have achieved reasonable success in predicting bulk properties (e.g., formation energy, bandgap) [25, 26, 27]. However, these hand‐crafted features often fail to capture the subtle, topology‐dependent variations in surface electronic structures that define the work function. More recently, Graph Neural Networks (GNNs) have demonstrated the ability to learn representations directly from crystal graphs [28, 29, 30, 31].

Recent machine‐learning studies have substantially advanced data‐driven prediction of work functions and related surface properties. Roy et al. applied machine‐learning models to work‐function prediction for two‐dimensional MXenes [6], while Palizhati et al. combined high‐throughput DFT calculations with convolutional neural networks to predict surface properties of intermetallic compounds [24]. Schindler et al. further integrated high‐throughput surface enumeration with machine learning to identify stable surfaces exhibiting extreme work functions [32]. More recently, Shi et al. introduced a surface‐emphasized multi‐task framework for predicting multiple properties of magnesium intermetallic surfaces [33], whereas Hsu et al. developed FIRE‐GNN, a force‐informed graph neural network designed for rapid and accurate prediction of surface properties [31]. Collectively, these studies demonstrate the effectiveness of descriptor‐based and graph‐based learning for surface‐property prediction and materials screening. However, they generally formulate the work function or related surface properties as direct scalar prediction targets and do not explicitly learn the complete spatial electrostatic‐potential landscape as an intermediate physical modality.

Despite these advances, a fundamental challenge persists: deep learning models are notoriously “data‐hungry”, requiring thousands of labeled examples to generalize, whereas high‐fidelity surface‐property datasets are scarce and costly to generate. Standard supervised learning on small datasets inevitably leads to overfitting, where the model memorizes specific training samples rather than learning the underlying physics [34, 35, 36]. To resolve this “data scarcity” dilemma, we draw inspiration from the recent success of multi‐modal contrastive learning (e.g., CLIP) [37, 38]. In this work, we propose a novel framework that treats the material's crystal structure and its plane‐averaged electrostatic potential (PAEPs) as two distinct but physically coupled modalities. Unlike traditional scalar regression, which compresses the rich electronic information into a single number (Φ), our model learns to align the vector representation of the 3D crystal graph with that of its 1D potential landscape [32, 33, 39].

In this work, we demonstrate that this cross‐modal alignment strategy enables the encoder to capture subtle topological features governing surface electronics, which are often overlooked in direct scalar supervision. Validated against a diverse library of 2D materials, our model exhibits robust predictive performance across wide regions of the periodic table, accurately capturing not just the work function values but also the statistical morphology of the electrostatic potential landscape. Furthermore, attention mechanism analysis reveals that the model autonomously “rediscovers” chemical intuition, effectively distinguishing the distinct electronic contributions of surface‐passivating species versus bulk‐shielded atoms. Crucially, we show that the learned representations internalize fundamental physical constraints, generating potential profiles that remain topologically consistent even in error regimes. This methodology establishes a physically interpretable foundation for accelerating the screening of 2D materials for next‐generation electronics.

## 2 Results

Before evaluating the predictive performance, we first outline the architecture of our Graph‐Potential Cross‐Modal Contrastive Learning framework, termed **StructPot‐CLR (Structure‐Potential Contrastive Learning Representation)**. As illustrated in Figure 1a, the model operates on a dual‐stream architecture: a 1D Convolutional Neural Network (CNN) encodes the Plane‐Averaged Electrostatic Potential (PAEP) landscape, while a specialized Crystal Encoder processes the atomic structure. To capture the subtle geometric variations governing surface electronics, the Crystal Encoder (Figure 1b) integrates a message‐passing mechanism with a Weighted Self‐Attention (W‐SA) block and rotationally equivariant layers (e3nn). This hybrid design ensures that the learned representations are not only sensitive to local coordination environments but also respectful of global rotational symmetries. During training, StructPot‐CLR optimizes a symmetric cross‐entropy loss to maximize the diagonal elements of the similarity matrix, effectively forcing the structural and potential representations of the same material to align within a high‐dimensional latent space.

**FIGURE 1:**
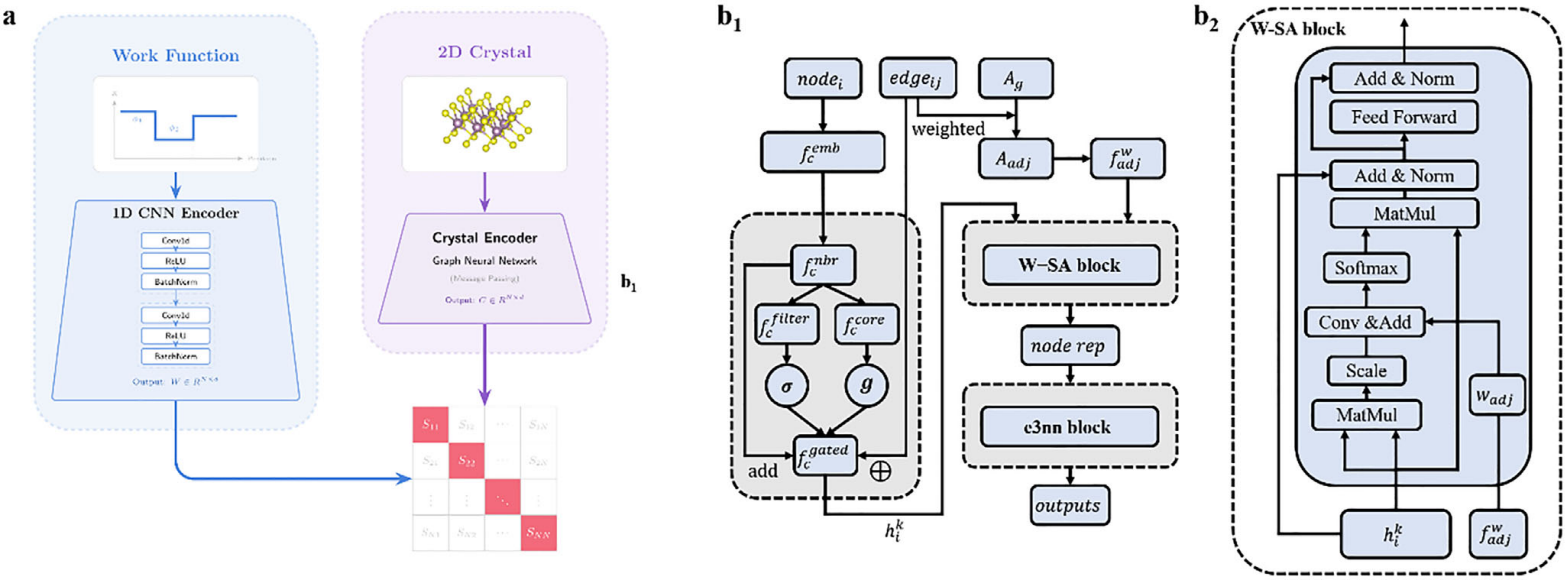
Schematic illustration of the StructPot‐CLR framework architecture. (a) The dual‐stream contrastive learning pipeline designed to align physical structures with electronic signatures. The framework consists of a 1D CNN encoder for the Plane‐Averaged Electrostatic Potential (PAEP) landscape and a Graph Neural Network (GNN) encoder for the 2D crystal structure. The model is trained to maximize the diagonal elements of the similarity matrix (S_NN_), forcing the structural and potential representations of the same material to converge in the high‐dimensional latent space. (b) Detailed architecture of the Crystal Encoder. (b1) The graph interaction layer, which processes atomic node features ($f_{c}^{\mathrm{emb}}$​), edge attributes (edge_ij_), and local geometry (A_g_) through a gated message‐passing mechanism ($f_{c}^{\mathrm{nbr}}$​) to update node representations. (b2) The internal structure of the Weighted Self‐Attention (W‐SA) block, which integrates multi‐head attention mechanisms with rotationally equivariant layers (e3nn block) to capture both long‐range dependencies and geometric symmetry constraints.

This alignment strategy establishes a shared semantic space where crystal structures and electronic potentials are mutually indexable. Figure 2 demonstrates the inference and retrieval capabilities enabled by this pre‐training. By inputting a target potential profile (query), the framework can identify candidate crystal structures from the database that best match the desired electronic topography (Top‐K Results). This process confirms that the model has successfully decoupled and re‐associated the “form” (crystal lattice) with the “function” (electrostatic potential). For the specific task of work function prediction, we utilize this pre‐aligned latent space to extract features that are intrinsically physically consistent. Unlike traditional supervised learning that maps structure directly to a scalar label, our approach ensures that the model's predictions are grounded in a deep understanding of the full potential landscape, thereby underpinning the physical interpretability discussed in the subsequent sections.

**FIGURE 2:**
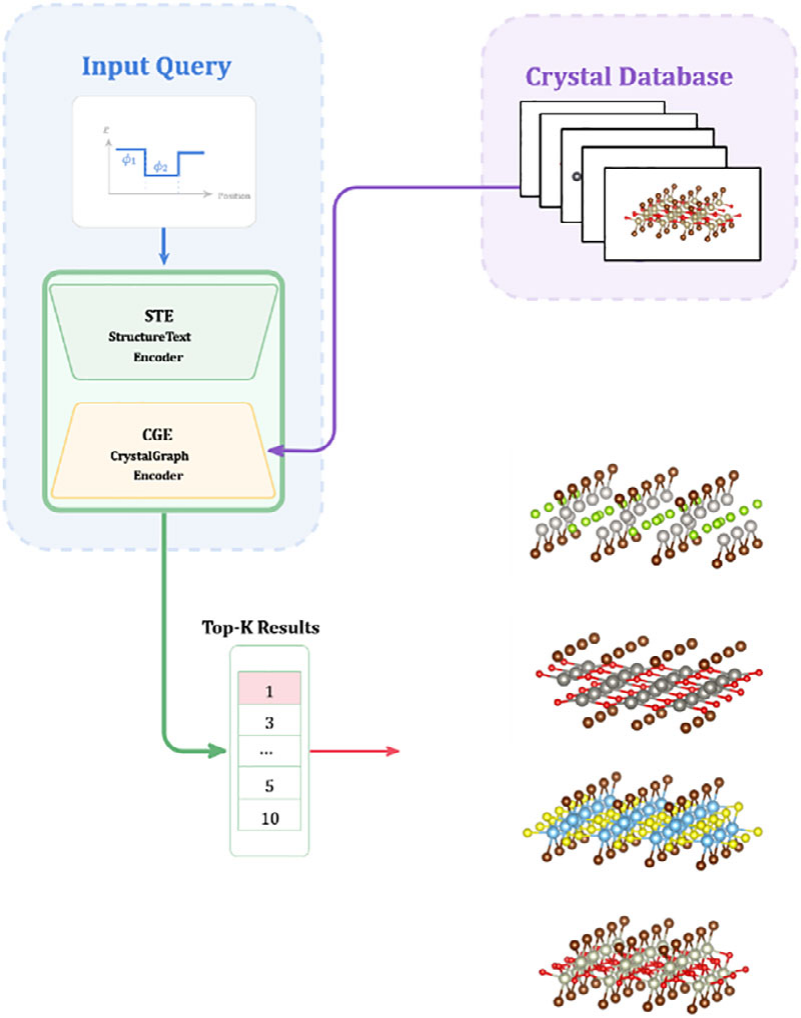
Cross‐modal retrieval and inverse screening workflow enabled by the pre‐aligned latent space. The diagram demonstrates the model's capability to map “function” back to “form.” A target electrostatic potential profile (Input Query), characterized by specific work function features (e.g., barrier heights $\phi_{1}$, $\phi_{2}$), is encoded and compared against a massive library of 2D material embeddings (Crystal Database). By calculating cosine similarities in the shared latent space, the model identifies and retrieves the Top‐K candidate crystal structures that are most likely to exhibit the desired electronic potential morphology, facilitating potential‐contour‐based inverse material design.

Our contrastive learning framework demonstrates robust work function prediction capabilities on a diverse dataset comprising 1,896 2D materials [40]. The model achieved strong correlations between predicted and ground‐truth values across multiple statistical and physical descriptors, indicating that it has successfully learned the underlying structure‐property relationships. To systematically evaluate the model's performance, we conducted three complementary analyses: (1) element‐resolved projection error distribution and property prediction accuracy, (2) elemental attention mechanism allocation, and (3) physical analysis of the learned representations [41].

### 2.1 Element‐Resolved Projection and Property Prediction Analysis

To assess the generalizability and predictive reliability of our model, we first evaluated the final work function prediction accuracy on the held‐out test set. As shown in Figure 3a, the predicted work functions exhibit strong agreement with DFT references, demonstrating robust regression performance across diverse 2D materials.

**FIGURE 3:**
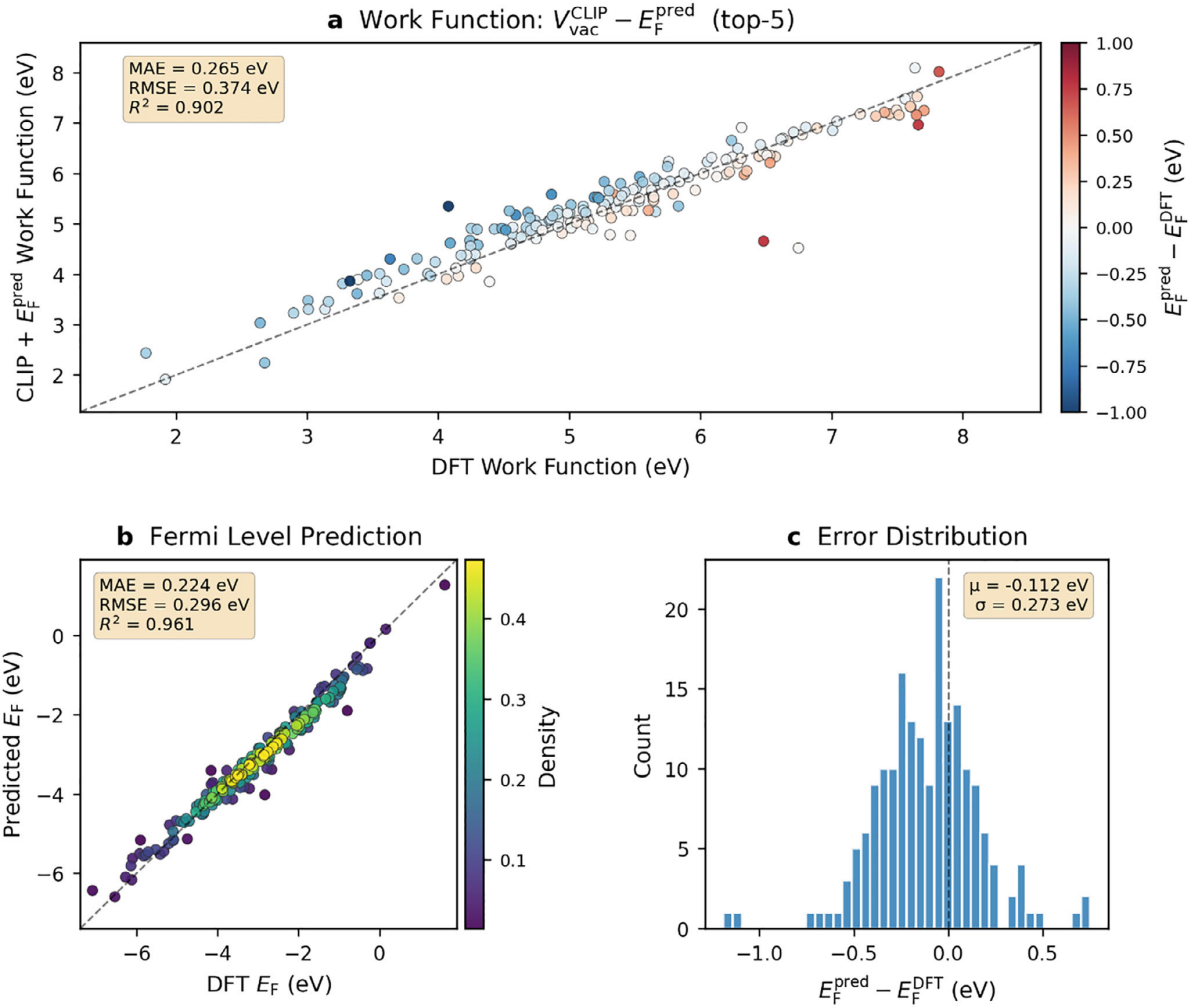
Quantitative evaluation of the work function and intermediate Fermi level predictions on the held‐out test set. (a) Scatter plot comparing the final predicted work function against the DFT ground truth. The predicted work function is strictly derived via the relation **Φ  = *E_vac_*  − *E_Fermi_***, where the vacuum level (***E_vac_***) is extracted from the potential plateau via the cross‐modal contrastive branch, and ***E_Fermi_*** is generated by the auxiliary regression head. The model achieves high predictive accuracy with a Mean Absolute Error (MAE) of 0.265 eV and an R^2^ of 0.902. The gray dashed line indicates the ideal y  =  x parity. (b) Scatter plot of the predicted Fermi level (***E_Fermi_***) versus the DFT‐calculated (***E_Fermi_***, confirming the robust performance of the auxiliary multi‐layer perceptron (MLP) head (MAE = 0.224 eV, R^2^ = 0.961). (c) Histogram illustrating the distribution of prediction errors for the Fermi level. The distribution is narrowly centered near zero (μ  =   − 0.112 eV, σ  =  0.273 eV), indicating that the auxiliary energy reference prediction is highly stable without severe systematic bias.

Crucially, rather than substituting the work function with an absolute vacuum level, our pipeline strictly derives the final observable as Φ  = *E_vac_*  − *E_Fermi_*. As summarized in Table 1 and Figure 3, the model achieves highly accurate predictions for the actual work function on the held‐out test set, yielding a Mean Absolute Error (MAE) of 0.265 eV and an R^2^ of 0.902. Furthermore, the high Pearson correlation (*r*  =  0.952) and Spearman rank correlation (ρ  =  0.927) coefficients demonstrate strong linear consistency and exceptional ranking capability, validating the framework's reliability for high‐throughput materials screening. This performance is supported by the robust extraction of the vacuum plateau via the PAEP branch and the concurrent prediction of the Fermi‐level reference via an auxiliary regression head.

**TABLE 1:** Predictive performance metrics for the work function (Φ  = *E_vac_*  − *E_Fermi_*) on the testing set. The table summarizes the accuracy (MAE, RMSE, *R* ^2^) and the linear/rank correlations (Pearson *r*, Spearman ρ) of the model predictions against DFT ground truths, validating its robust applicability for high‐throughput screening.

Metric	Value
MAE	0.265 eV
RMSE	0.374 eV
*R* ^2^	0.902
Pearson *r*	0.954 (*p* = 7 × 10^−^^9^ ^9^)
Spearman *ρ*	0.932 (*p* = 1 × 10^−83^)

Figure S1 shows that the predicted Skewness and Kurtosis values cluster closely around the ideal line (y = x). The high predictive accuracy for Skewness (*R* ^2^ = 0.9824) and Kurtosis (*R* ^2^ = 0.9808) demonstrates that the model reliably captures the asymmetry and peakedness of the potential distribution associated with surface charge rearrangements and dipole layers [42]. Even without explicit emphasis on numerical differences—unlike the direct constraints typically applied in the loss functions of supervised learning—the model automatically maintains high robustness, accurately anchoring the absolute amplitude and dynamic range of the potential landscape in the energy dimension. These results confirm that the model, trained via contrastive learning, has transcended mere scalar regression to successfully internalize a profound understanding of the microscopic variations within the electrostatic potential landscape [43].

### 2.2 Benchmarking and Ablation Analysis

To rigorously validate the architectural advantages of the StructPot‐CLR framework, we benchmarked its performance against representative machine learning baselines and systematically investigated the contribution of individual components. As summarized in Figure 4, the proposed framework achieves superior predictive accuracy compared with conventional structure‐based approaches, while ablation experiments reveal the essential roles of electrostatic potential encoding and cross‐modal contrastive alignment. Compared to standard graph neural networks (e.g., CGCNN, ALIGNN) and descriptor‐based ensemble models evaluated under the identical data split, our cross‐modal framework achieves state‐of‐the‐art predictive accuracy for the work function. Furthermore, ablation experiments confirm that both the PAEP encoder and the contrastive pretraining objective are indispensable; omitting the PAEP modality or contrastive alignment leads to substantial performance degradation, confirming that the performance gain originates from physical cross‐modal representation learning rather than increased model complexity. These results quantitatively demonstrate that explicitly aligning the 3D crystal graph with its 1D electrostatic potential landscape provides a critical advantage over conventional direct scalar regression.

**FIGURE 4:**
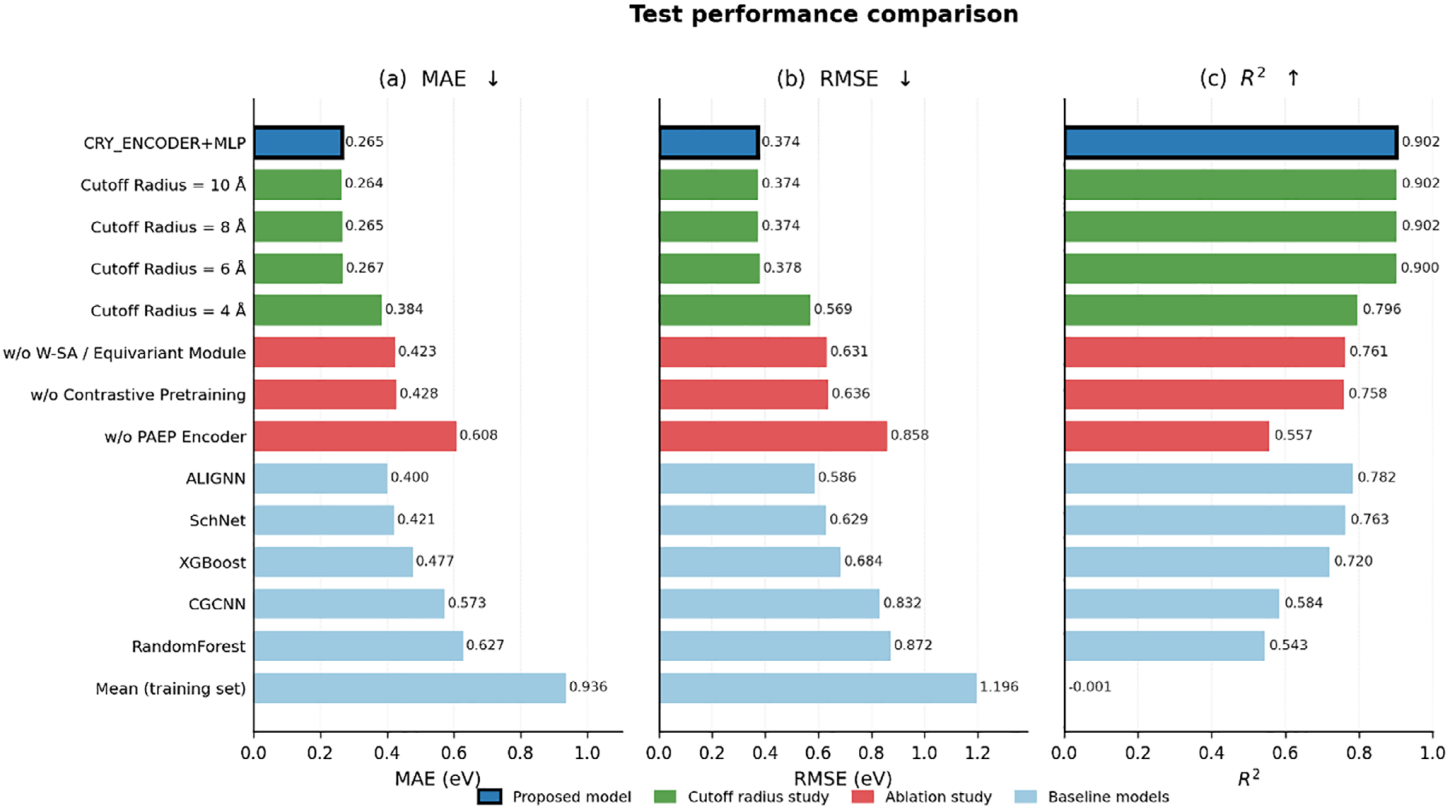
Benchmarking and ablation analysis of the cross‐modal contrastive learning framework (StructPot‐CLR) for work function prediction. Performance on the held‐out test set is compared using three evaluation metrics: (a) mean absolute error (MAE; lower is better), (b) root mean square error (RMSE; lower is better), and (c) coefficient of determination (R^2^; higher is better). The proposed CRY_ENCODER+MLP model, trained through cross‐modal contrastive pretraining between crystal structures and plane‐averaged electrostatic potential (PAEP) landscapes, is evaluated against a non‐learning mean baseline, descriptor‐based machine learning models (RandomForest and XGBoost), and graph neural networks (CGCNN, ALIGNN, and SchNet). The mean baseline assigns every test sample the mean work function calculated exclusively from the training set. Ablation studies quantify the contributions of contrastive pretraining, PAEP encoding, and the W‐SA/equivariant module, while cutoff‐radius experiments assess the influence of the structural neighborhood range. Overall, the results show that explicitly aligning structural and electrostatic representations substantially improves work function prediction over conventional structure‐to‐property regression approaches.

### 2.3 Attention Mechanism Analysis

To gain deeper insights into the model's decision‐making mechanism, we systematically analyzed the distribution of attention weights across 1449 metal‐nonmetal compounds. Figure 5 illustrates the surface occupancy rates of non‐metal elements and the metal‐to‐nonmetal attention allocation ratios across different compound types (oxides, sulfides, selenides, tellurides, chlorides, bromides, and iodides).

**FIGURE 5:**
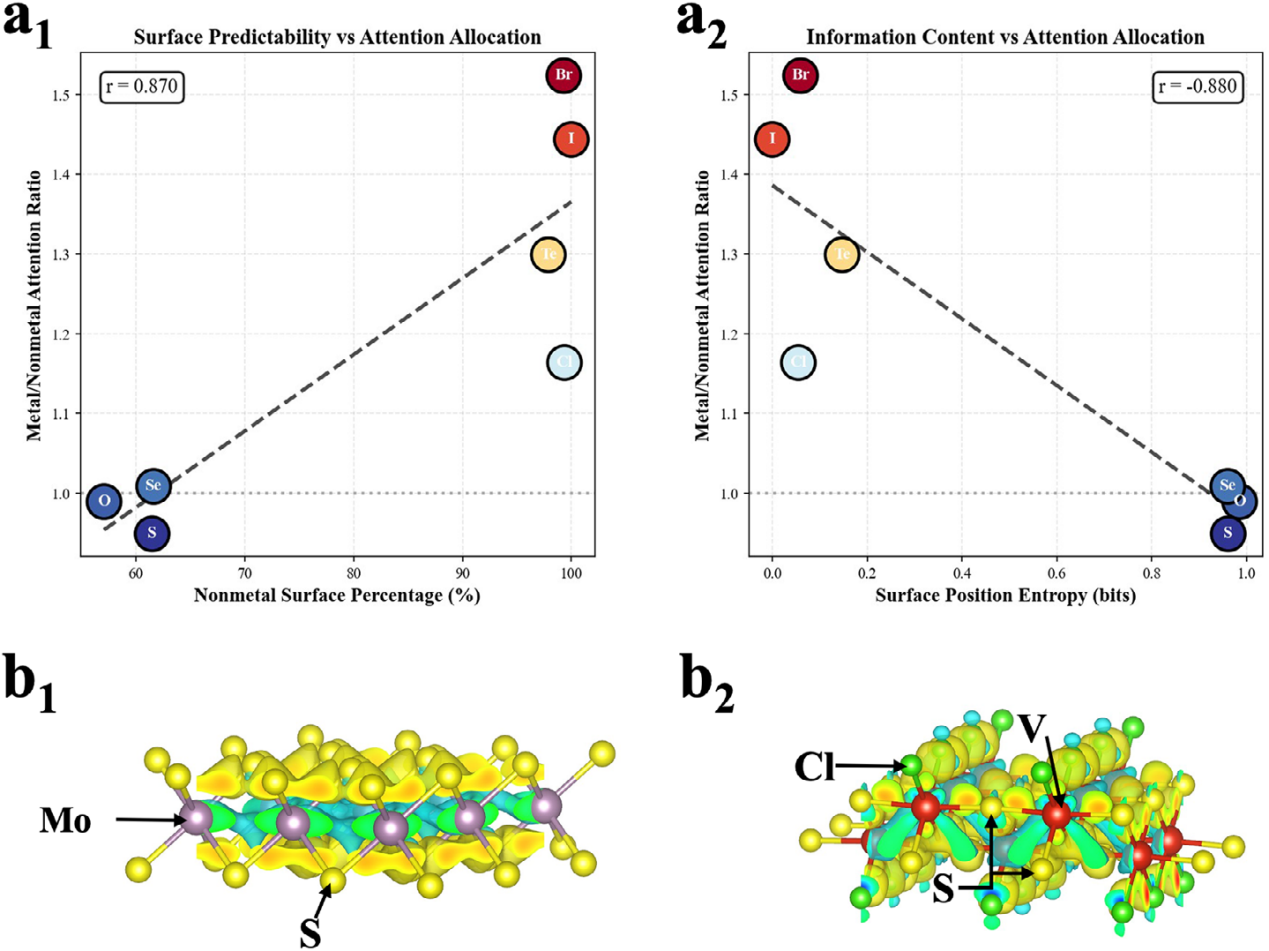
Attention mechanism analysis revealing elemental combination effects. (a1 and a2) Correlation analysis of the metal/non‐metal attention ratio with non‐metal surface occupancy (%) and surface position entropy (bits), respectively. Data are derived from posterior statistics of 1449 samples. (b1 and b2) Comparison between differential charge density and attention allocation for representative systems. (b1) The MoS_2_ system, where surface S atoms exhibit significant electron accumulation; (b2) the V_2_Cl_2_S_2_ system, illustrating the distinct charge distributions of inner S atoms versus surface Cl atoms under specific layered stacking.

From the perspective of model interpretability, attention weights intuitively reflect the neural network's preference for utilizing information from specific nodes within the crystal graph structure [28]. Although high attention weights do not directly equate to causal discovery, they imply that the features of those specific elemental nodes carry higher signal strength within the model's decision path [44]. By parsing these allocation patterns, we can understand how the model dynamically modulates its “Attention Focus” in response to varying chemical environments when predicting work functions. The statistical results in Figure 5a reveal that halogen elements (Cl, Br, I) exhibit an extremely high propensity for surface occupation in 2D materials, with iodide samples reaching a surface occupancy of 100%. In contrast, the distribution of chalcogens (O, S, Se) is more balanced. This discrepancy aligns well with chemical intuition: halogen atoms tend to occupy outer sites to act as surface passivators, whereas chalcogens can serve both as skeletal components and surface passivators [45]. The scatter plots further demonstrate a gradient shift in the model's attention allocation: transitioning from a non‐metal dominance in sulfides (ratio < 1) to a metal dominance in halides (ratio > 1). Notably, for iodine and bromine, whose positions are highly deterministic, the model shifts its focus to the metal centers. This can be interpreted through the lens of information entropy: for S/Se atoms with high spatial uncertainty (high entropy), the model must allocate higher attention weights to resolve their specific coordination environments; conversely, for halogens that almost exclusively occupy the surface (low entropy), their geometric information is relatively redundant. Consequently, the model redirects its attention to the metal centers, which play a more decisive role in determining electronic structure variations [46]. To analyze the model's coupled understanding of “position” and “electronic state,” we dissected two case studies shown in Figure 5b. In the MoS_2_ system (Figure 5b1), S atoms are located on the outermost surface. The differential charge density map shows significant electron accumulation (yellow regions) on the surface S atoms due to their high electronegativity. Given the high sensitivity of the work function to the surface dipole layer, the model acutely captures this, assigning an exceptionally high attention weight to the surface S atoms (Attention Score ≈ 2.38), far exceeding that of the metal Mo (≈ 1.02) [10, 19]. This indicates that in pure sulfides, the model correctly identifies surface anions as the key drivers determining the potential barrier height. However, an intriguing reversal occurs in the VClS system (Figure 5b2). Although the S atom remains electronegative, it is “sandwiched” between V and Cl layers, functioning as an internal bridging atom, while Cl atoms occupy the outermost surface. In this configuration, the model's attention on S drops precipitously to approximately 0.32, while the focus is redistributed to the metal V (≈ 1.42) and surface Cl (≈ 1.09). This significant weight disparity (*S*_surface_ ≫ *S*_bulk_) strongly confirms that the model is not merely mechanically memorizing that “sulfur is important”. Instead, it possesses a profound spatial awareness: it understands that atoms located in the bulk are subject to geometric screening, making their direct contribution to the vacuum level and surface barrier significantly smaller than that of surface atoms [47, 48].

### 2.4 Learned Representations of Electronic Structure

To verify that the model has learned physically meaningful representations rather than spurious correlations, we analyzed the predicted work function profiles for outlier samples and compared them with ground‐state DFT calculations.

To deeply investigate the model's physical representation capabilities and sources of error, we deliberately selected samples with large prediction deviations to examine their reconstructed plane‐averaged electrostatic potential profiles. This physical quantity acts as a bridge connecting crystal structure to the work function; its spatial profile not only defines the barrier height for electron spillover but also precisely depicts the complex electrostatic potential fluctuations and microscopic charge distribution characteristics within the lattice.

As shown in Figure 6, even in cases where prediction deviations occur, the model demonstrates remarkable sensitivity to physical information. The oscillatory features of the potential curve correspond clearly to the layered arrangement of atomic planes—potential wells (minima) correspond to atomic nucleus positions, while potential barriers (maxima) correspond to interlayer gaps. Furthermore, the model exhibits an understanding of electron density topology through the curvature characteristics of the potential profile. According to the Poisson equation (∇^2^ *V*∝ − ρ), the second derivative of the potential function directly corresponds to the charge density distribution [49]. The predicted profiles not only reproduce the steep potential drop near atomic nuclei (high electron density regions) but also fit the gradual potential rise in interstitial regions (low electron density regions). This topological consistency indicates that, even for erroneous samples, the model has implicitly learned the modulation of local charge distribution by the atomic coordination environment directly from structural stacking information. Moreover, a closer inspection of Figure 6 reveals that although systematic deviations in amplitude may exist between the predicted potential well depths and ground truth within the material interior (bulk region), these deviations are primarily manifested in the “magnitude” of the energy scale rather than the “structure” of the spatial distribution. This estimation error in potential well depth directly leads to a drift in the Fermi level reference point. Since the work function is defined as the difference between the vacuum level and the Fermi level (Φ  = *E_vac_*  − *E_Fermi_*), even if the model correctly predicts the vacuum level (as shown by the plateaus on both sides), a misjudgment of the internal average potential depth results in a deviation in the final work function value. Crucially, this study employs a contrastive learning framework [50]. During training, the model was never explicitly provided with specific values for the vacuum level (*E_vac_*) or Fermi level (*E_Fermi_*) as intermediate supervisory signals, nor was it pre‐defined with the physical formula for the work function. Instead, the model was forced to rely solely on crystal structure information to autonomously explore and implicitly “derive” these two key physical quantities determining the work function. Consequently, the prediction deviation in absolute values likely stems from a systematic drift in the quantitative scaling of the internal potential depth, arising from the lack of strong supervision on absolute values. This specific error pattern, characterized by a correct geometric topology but a shifted energy amplitude, demonstrates that the model's prediction deviations are not chaotic random noise, but rather systematic drifts with clear physical attributions [51]. Specifically, it suggests that errors do not stem from a misunderstanding of crystal geometry leading to fundamental physical errors, but rather from quantitative estimation biases regarding the strength of electron screening effects. In other words, even when “making mistakes,” the model constructs a geometrically reasonable and topologically correct physical field, indicating that it has built a valid physical representation rather than merely completing a statistical fitting of scalar data.

**FIGURE 6:**
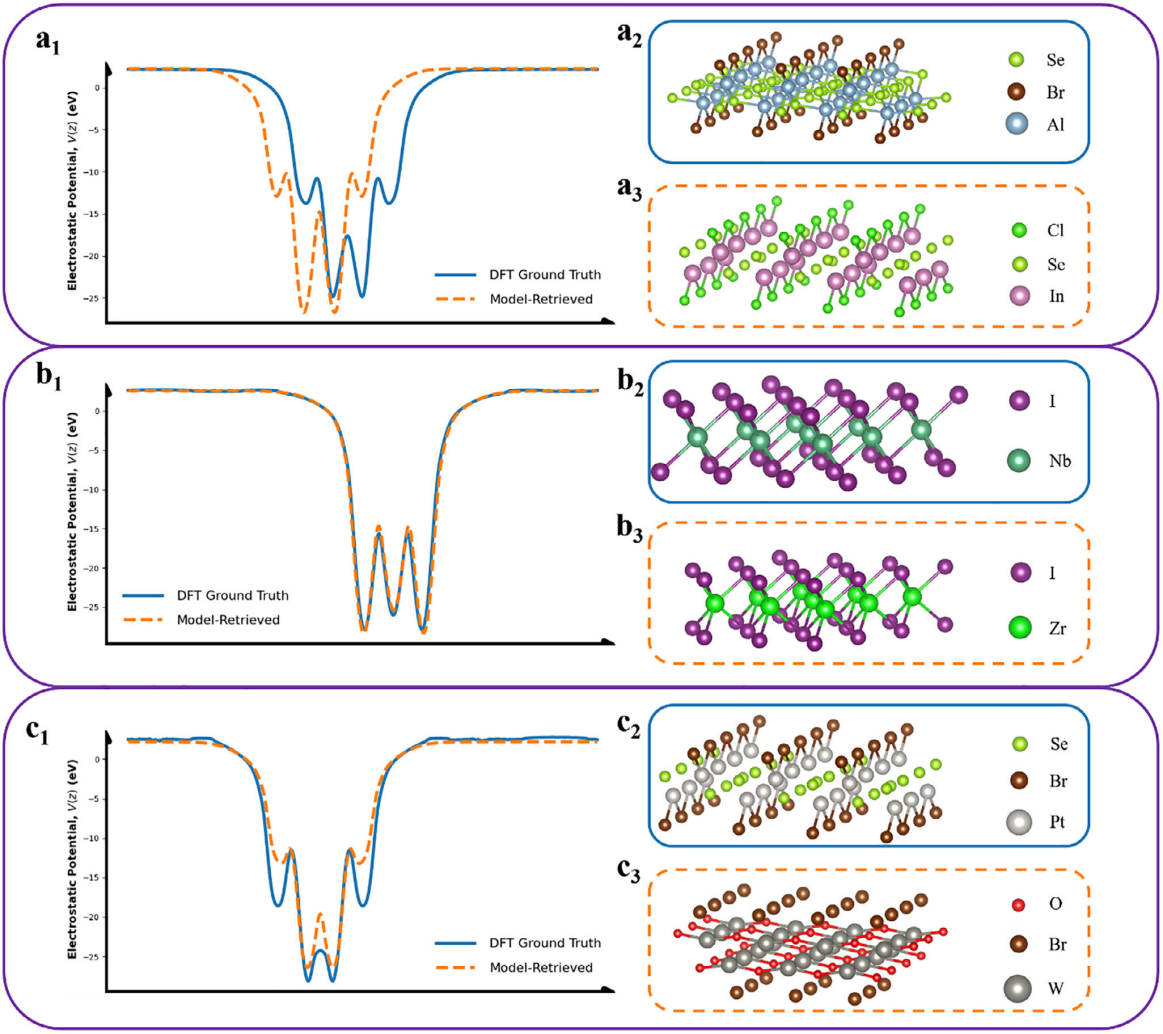
Revealing the model's physical representation capability through electrostatic potential profile analysis of outliers samples. Comparison of the Plane‐Averaged Electrostatic Potential (PAEP) along the surface normal direction between predicted values (orange) and ground‐truth Density Functional Theory (DFT) calculations (blue).

## 3 Methods

Our work is centered on a multi‐modal contrastive learning framework designed to predict the work function of 2D materials. The core principle is to learn a shared embedding space where the representations of a material's crystal structure and its corresponding PAEP are aligned. This is achieved by training two independent encoders—one for the crystal graph and one for the PAEP sequence—whose outputs are optimized to maximize the similarity between corresponding pairs while minimizing it for non‐corresponding pairs. The architecture is inspired by the CLIP framework, adapted for materials science applications.

### 3.1 Crystal Structure Encoder

The crystal structure encoder, *f*_cry_(·), is a deep graph neural network that maps a crystal graph G=(V,E) to a fixed‐dimensional vector representation $z^{\mathrm{cry}}\in R^{D}$[28]. The input features for each atom i∈V consist of its atomic number *a_i_*, and the input for each edge (i,j)∈E is derived from the interatomic distance *d_ij_* via a Gaussian radial basis function expansion [52].

For a crystal with *T* atoms, the initial node features are derived from an embedding layer: $h_{i}^{\left(0\right)}=E_{\mathrm{elem}}\;\left[a_{i}\right]$, where $E_{\mathrm{elem}}\in R^{V\times D_{\mathrm{elem}}}$ is a learnable embedding matrix for *V* unique elements. Interatomic distances are expanded using a Gaussian basis function to capture local environment information:

(1) $$e_{ij}=\;\exp\left(-\frac{{\left(d_{ij}-\mu \right)}^{2}}{\gamma^{2}}\right)$$

where **μ** and **γ** are fixed parameters defining the centers and widths of Gaussian filters over a cutoff distance of 8.0 Å. The robustness of the 8.0 Å cutoff was verified through a small‐scale ablation study (4.0, 6.0, 8.0, 10.0 Å). While a 4.0 Å cutoff led to performance degradation due to missing long‐range context, the model's performance effectively saturated beyond 6.0 Å. We maintained 8.0 Å as a conservative choice to safely encompass van der Waals and electrostatic screening effects while avoiding the computational overhead of overly dense graphs (e.g., at 10.0 Å).

Two CGCNN‐inspired layers update the node representations. The update for atom *i* at layer *l* is given by:

(2) $$h_{i}^{\left(l+1\right)}=h_{i}^{\left(l\right)}+\mathrm{Softplus}\left(\sum_{j\in N\left(i\right)}\sigma \left(z_{ij}^{\left(l\right)}\right)⊙g\left(z_{ij}^{\left(l\right)}\right)\right)$$

where N(i) is the set of neighbors of atom *i*, $z_{ij}^{\left(l\right)}=W_{f}^{\left(l\right)}\;\left[h_{i}^{\left(l\right)}∥h_{j}^{\left(l\right)}∥e_{ij}\right]+b_{f}^{\left(l\right)}$, and *g*(·) is an activation function (Softplus). The feature vector is split into two halves, with one processed by a sigmoid gate σ(·) that modulates the other half [53]. *W_f_* and **b**_*f*_ are learnable parameters.

Additionally, a local geometric feature vector $x_{i}^{\mathrm{extra}}\in R^{363}$ is computed for each atom, encoding the distances and angles between atom *i*, its 11 nearest neighbors *j*, and their respective 11 nearest neighbors *k*  [54].The node features are then fused with the local geometric features $x_{i}^{\mathrm{extra}}$ and processed by a multi‐head self‐attention Transformer [55]. The attention score between atoms *i* and *j* is modified with a learnable adjacency bias [56]:

(3) $$\alpha_{ij}=\frac{\exp\left(\frac{\left(q_{i}^{T}k_{j}\right)}{\sqrt{d_{k}}}+B_{ij}\right)}{\sum_{k=1}^{T}\exp\left(\frac{\left(q_{i}^{T}k_{k}\right)}{\sqrt{d_{k}}}+B_{ik}\right)}\;$$

where **q**_*i*_ and **k**_*j*_ are the query and key vectors, and the bias $B_{ij}=\phi \left(e_{ij}\right)$ is computed by a small MLP ϕ from the Gaussian distance features.

The final node representations from the Transformer are projected to a 640‐dimensional space and then aggregated into a single graph‐level embedding using *O*(3)‐equivariant layers from the e3nn library [57]. To incorporate geometric directionality, these equivariant layers additionally take the relative position vectors ${\vec{r}}_{ij}={\vec{r}}_{i}\;-{\vec{r}}_{j}$ (via spherical harmonics) as input attributes together with the scalar distance embeddings [58]. The features are split and processed through parallel equivariant linear layers operating on irreducible representations (irreps) of types 0e (scalar) and 1o (pseudo‐vector), ensuring rotational invariance. The resulting atomic features are summed to produce the final crystal embedding **z** ^cry^.

### 3.2 Plane‐Averaged Electrostatic Potential Encoder

The PAEP encoder, *f*_prof_(·), is a 1D Residual Network (ResNet) that processes the PAEP sequence $w\in R^{L}$ to produce an embedding $z^{\mathrm{prof}}\in R^{D}$[53, 59]. The architecture consists of a convolutional stem followed by four residual stages. Each residual block is defined as:

(4) $$w^{\left(l+1\right)}=\;\mathrm{ReLU}\left(F\left(w^{\left(l\right)},\left\{W_{i}^{\left(l\right)}\right\}\right)+w^{\left(l\right)}\right)$$

where F represents two 1D convolutions with BatchNorm and ReLU. The channel depth is progressively increased (64, 128, 256, 512) and spatial dimensions are reduced via strided convolutions between stages. A final global average pooling layer and a linear projection produce the embedding **z** ^prof^.

Given a batch of *N* (crystal, PAEP) pairs, we compute their embeddings ${\left\{z_{i}^{\mathrm{cry}},z_{i}^{\mathrm{prof}}\right\}}_{i=1}^{N}$. These are passed through linear projection heads and L2‐normalized to obtain ${\left\{{\hat{p}}_{i}^{\mathrm{cry}},{\hat{p}}_{i}^{\mathrm{prof}}\right\}}_{i=1}^{N}$. The similarity between the *i*‐th PAEP and the *j*‐th crystal is the cosine similarity $s_{ij}={\left({\hat{p}}_{i}^{\mathrm{prof}}\right)}^{T}{\hat{p}}_{j}^{\mathrm{cry}}$.

We minimize a symmetric cross‐entropy loss to optimize the mutual information between the two modalities. This objective explicitly aligns the structural representations with their corresponding electronic landscapes in the shared latent space, ensuring that chemically similar structures map to similar potential profiles.

(5) $$L=-\frac{1}{2N}\sum_{i=1}^{N}\left[\log\frac{\exp\left(s_{ii}/\tau \right)}{\sum_{j=1}^{N}\exp\left(s_{ij}/\tau \right)}+\log\frac{\exp\left(s_{ii}/\tau \right)}{\sum_{j=1}^{N}\exp\left(s_{ji}/\tau \right)}\right]$$

where τ is a learnable temperature parameter that scales the logits [37].

The model was trained on a dataset of 1,896 2D materials (exclusively monolayer slabs, including 17.2% Janus structures with asymmetric configurations), split into 80% for training, 10% for validation, and 10% for testing. We used the AdamW optimizer with a learning rate of 1 × 10^−4^ and a cosine annealing schedule. The embedding dimension *D* was set to 384, and the batch size was 64 [54]. Training was conducted for 1000 epochs, with the model checkpoint corresponding to the lowest validation loss being saved for inference.

### 3.3 Auxiliary Regression Head and Final Observable Computation

Following the cross‐modal contrastive alignment, the global crystal representation (**z** ^cry^) extracted by the *O*(3)‐equivariant encoder acquires rich electrostatic and topological information. To predict the final work function with strict physical consistency, **z** ^cry^ is routed into a lightweight multi‐layer perceptron (MLP) acting as an auxiliary regression head to explicitly predict the Fermi level ***E***_***Fermi***_). Concurrently, the absolute vacuum level (***E***_***vac***_) is extracted directly from the asymptotic plateau of the PAEP profile processed by the 1D ResNet branch. Rather than performing a direct end‐to‐end scalar approximation, the final work function is deterministically computed via the thermodynamic relation **Φ**  = ***E***_***vac***_  − ***E***_***Fermi***_. This architectural design rigorously enforces physical constraints on the deep learning pipeline, ensuring the interpretability of the final outputs.

To substantiate the computational efficiency, we benchmarked StructPot‐CLR against standard DFT. While calculating the PAEP for a typical 2D unit cell via DFT requires several CPU hours, our model achieves an inference latency of merely 0.119 ms per structure on a single GPU (throughput > 8300 samples/s). This ∼10^7^‐fold acceleration effectively enables ultra‐fast, high‐throughput materials screening.

It is worth noting that standard cross‐modal contrastive frameworks (e.g., CLIP) often suffer from a ‘relational understanding deficiency,’ struggling to distinguish samples with identical compositions but different spatial relations. In the context of materials science, this translates to the challenge of differentiating subtle polymorphs. Our StructPot‐CLR framework mitigates this limitation through two aspects: architecturally, the O(3)‐equivariant crystal encoder preserves rigorous directional symmetries to prevent spatial feature collapse; algorithmically, the presence of identical‐formula structural variants in the dataset acts as implicit ‘hard negatives’ during the InfoNCE optimization, forcing the model to transcend lazy compositional shortcuts and learn fine‐grained topological reasoning.

Importantly, the contrastive branch is trained using the complete discretized PAEP profile rather than the scalar work function as its curve‐level training modality. During inference, the embedding of a query crystal structure is compared with the PAEP embeddings in the reference library using cosine similarity. The five PAEP profiles with the highest similarities are retrieved, and their vacuum plateau values are arithmetically averaged to obtain *E*_vac_. The use of the five most similar profiles reduces the sensitivity of the estimated vacuum level to any individual retrieval. In parallel, the contrastively pretrained crystal representation is passed to an auxiliary multilayer perceptron to predict *E*_Fermi_. The final work function is not directly predicted by the contrastive branch but is deterministically derived as Φ  =  *E*_vac_ −  *E*_Fermi_.

### 3.4 Dataset Curation and Splitting Strategy

The ground‐truth PAEP labels and corresponding 3D crystal structures for the 1896 diverse monolayer 2D materials were directly sourced from the MatHub‐2d database [36]. For reproducibility, the underlying high‐throughput DFT calculations in this database were performed using the GGA‐PBE exchange‐correlation functional with a plane‐wave energy cutoff of 500 eV, and a generous vacuum padding of 30 Å was applied along the *z*‐direction to strictly eliminate spurious periodic electrostatic interactions. A comprehensive statistical overview of the elemental coverage and property distributions of this dataset is provided in Figure S2.

To evaluate the model's generalizability, the dataset was partitioned into training (80%), validation (10%), and testing (10%) sets via a random splitting strategy. To rigorously rule out the possibility of structural or compositional data leakage—a common pitfall in materials informatics—a post‐hoc leakage analysis was conducted (Figure S3). The analysis confirmed a remarkably low compositional overlap (16.4%) between the training and testing sets. This verifies that the model's high predictive accuracy on the held‐out test set is driven by its genuine capability to map physical topologies, rather than merely memorizing homologous chemical formulas.

## 4 Conclusion

Addressing the dual challenges of high‐precision data scarcity and complex physical mechanisms in the field of surface science for 2D materials, this study proposes a Graph‐Potential Surface Cross‐Modal Contrastive Learning framework. Distinct from traditional “structure‐to‐scalar” mapping paradigms, this approach forces the alignment of 3D crystal geometric information with 1D electrostatic potential landscapes (PAEP) within a high‐dimensional latent space. This strategy successfully imbues the deep learning model with significant physical interpretability through a purely data‐driven training process. In‐depth experimental analysis demonstrates that the model exhibits physical intuition transcending simple statistical fitting. Its attention mechanism autonomously identifies “functional regions,” effectively distinguishing the dominant role of surface passivation atoms from the geometric screening effects of bulk atoms. This observation aligns highly with the surface dipole layer theory in solid‐state physics. Simultaneously, by evaluating the work function as the strict energy difference between the PAEP‐derived vacuum level and the auxiliary predicted Fermi level (Φ  = *E_vac_*  − *E_Fermi_*), the model guarantees the physical consistency of the final prediction while reconstructing the topological morphology of the potential with high precision. Building upon this established framework, which combines high precision with physical interpretability, future work will extend deeply into three strategic directions:

First, given the model's demonstrated sensitivity to surface dipole moments, it can be directly extended to predict band alignment in van der Waals heterostructures. By accurately assessing interfacial charge transfer and Schottky barrier heights, this approach offers potential insights into solving the long‐standing challenge of Fermi level pinning at heterostructure interfaces. Second, leveraging the established “structure‐potential surface” mapping, we intend to invert the design workflow. By utilizing guided generative models to inversely search for or generate crystal structures that satisfy specific criteria, we aim to achieve a paradigm shift from traditional “property screening” to “targeted design”. Finally, capitalizing on the model's sensitivity to prediction uncertainty (manifested through potential surface reconstruction errors), this framework is well‐suited for integration into closed‐loop active learning systems. By autonomously identifying novel chemical spaces where physical mechanisms remain unclear for prioritized calculation, this approach promises to significantly expedite the discovery of high‐performance anti‐corrosion coatings and next‐generation nano‐electronic materials with minimal computational cost.

## Funding

No funding was received for this work.

## Conflicts of Interest

The authors declare no competing financial interests or personal relationships that could have appeared to influence the work reported in this paper.

## Supporting information




**Supporting File**: advs77166‐sup‐0001‐SuppMat.docx.

## Data Availability

The complete source code, pre‐trained model weights, and inference scripts for the StructPot‐CLR framework are publicly hosted on GitHub at https://github.com/Wanhaoyu0715/StructPot‐CLR to facilitate reproducibility. The codebase is implemented using PyTorch, e3nn, and PyMatgen. The original 2D materials structural data and corresponding PAEP labels analyzed during this study are publicly available from the MatHub‐2d database.
