# Supplementary material for: Learning Work Function via Implicit Reasoning on Electrostatic Potential Landscapes
Source: Adv Sci (Weinh). 2026 Aug 18:e77166. Online ahead of print. doi: 10.1002/advs.77166 (PMC13483140; doi:10.1002/advs.77166)
Supplement: Supplementary file 1 [file ADVS-9999-e77166-s001.docx]

**Supplementary Materials**

**Learning Work Function via Implicit Reasoning on Electrostatic Potential Landscapes**

Haoyu Wan^a,§^, Yue Wu^a, §^, Tianhao Su^a^, and Deng Pan^a,b,∗^

^a^ Materials Genome Institute, Shanghai University, Shanghai, 200444, PR China

^b^ Ministry of Education Key Laboratory of Silicate Cultural Relics Conservation, Shanghai University, Shanghai, 200444, PR China

^§^ These authors contributed equally to this work.

^∗^ Corresponding authors.

Haoyu Wan: ^∗^ for correspondence (journal submission and communication), E-mail: Wanhaoyu0715@gmail.com;

Deng Pan: ^∗^ senior corresponding author (academic supervision and design), E-mail: DPan_MGI@shu.edu.cn.

E-mail addresses for other authors: repeatsongyue@gmail.com (Yue Wu); thsu0407@gmail.com (Tianhao Su).

**Funding**

No funding was received for this work.

**Conflict of Interest**

The authors declare no competing financial interests or personal relationships that could have appeared to influence the work reported in this paper.

**Supplementary Figures and Graphs**

**Figure S1. Parity plots of statistical descriptors of the plane-averaged electrostatic potential (PAEP) landscapes.** Comparison of the descriptor values calculated from DFT-derived PAEP profiles (ground truth) with those obtained from the PAEP profiles reconstructed by StructPot-CLR for the held-out test set (*N* = 191): (a) skewness, which characterizes the asymmetry of the potential distribution (*R*² = 0.9824), and (b) kurtosis, which characterizes its peakedness (*R*² = 0.9808). Each blue point represents an individual 2D material, and the red dashed line denotes ideal agreement (*y* = *x*). The close correspondence between the predicted and DFT-derived values demonstrates that the model accurately preserves the higher-order statistical morphology of the electrostatic potential landscapes.

 **Figure S2. Statistical overview of the 1,899 2D materials in the dataset.** (a) Element coverage mapped onto the periodic table. The color gradient indicates the number of structures containing each specific element, demonstrating broad chemical diversity with a high concentration of chalcogens and halogens typical for surface-passivated 2D materials. (b) Distribution histograms of the ground-truth Work Function (WF) and Fermi Level ($\boldsymbol{E}_{\boldsymbol{Fermi}}$). The broad energy spans (WF: $\mu$=5.30 eV, $\sigma$ =1.34 eV; $\boldsymbol{E}_{\boldsymbol{Fermi}}$: $\mu=-3.12$ eV,$\sigma$ =1.51 eV) ensure that the model learns robust physical mappings across diverse electronic states rather than memorizing a narrow numerical band.

**Figure S3. Mitigation of compositional data leakage through cross-modal contrastive learning.** The figure illustrates two representative scenarios of homologous samples (identical chemical formulas) distributed across the training and testing sets. (Top) Two In_2_Se_2_ polymorphs exhibit distinct structural prototypes, resulting in drastically different planar-averaged electrostatic potential (PAEP) profiles ($\Delta\text{WF}=5.156$ eV). This fundamentally prevents the model from exploiting simple elemental composition shortcuts. (Bottom) Two MoS_2_ stacking variants display highly identical PAEP profiles ($\Delta\text{WF}=0.105$ eV). In both scenarios, the symmetric InfoNCE loss forces the model to perform fine-grained bidirectional retrieval based on exact spatial geometry rather than mere chemical formulas. Consequently, these homologous pairs naturally serve as intrinsic "hard negatives" during pre-training, ensuring robust physical representation learning while circumventing lazy structural leakage.

**Table S1. Summary statistics of the work function distribution in the dataset.** Following data cleaning, three outlier samples were removed from the original dataset. The table presents the 1896 samples range (0.982 to 9.946 eV), mean (5.321 eV), and standard deviation (1.257 eV) of the Density Functional Theory (DFT) calculated work function values across the curated 2D materials dataset.

| **Statistic** | **Value** |
| --- | --- |
| Range | 0.982 to 9.946 eV |
| Mean | 5.321 eV |
| Standard deviation | 1.257 eV |

**Table S2. Work function prediction metrics across the training, validation, and test splits.** The dataset was partitioned using a purely random 80/10/10 split. The comparable Mean Absolute Error (MAE), Root Mean Square Error (RMSE), and coefficient of determination (R²) across the training (N=1519), validation (N=189), and testing (N=191) sets demonstrate that the model generalizes robustly to unseen crystal structures and does not suffer from severe overfitting.

| **Split** | **N** | **WF MAE** | **WF RMSE** | **WF R²** |
| --- | --- | --- | --- | --- |
| Train | 1519 | 0.207 | 0.268 | 0.963 |
| Val | 189 | 0.229 | 0.303 | 0.939 |
| Test | 191 | 0.269 | 0.381 | 0.898 |

**Table S3. Performance benchmark of the StructPot-CLR framework against representative machine learning baselines.** All models were trained and evaluated on the exact same dataset split to predict the final work function ($\boldsymbol{\Phi}$). The baselines encompass an elemental-feature Random Forest, standard Graph Neural Networks (SchNet, ALIGNN, CGCNN), and a direct scalar regression variant of our crystal encoder that omits the PAEP modality. The superior predictive accuracy (lowest MAE and RMSE, highest R²) of StructPot-CLR quantitatively highlights the indispensable advantage of integrating the intermediate electrostatic-potential modality.

| **Model** | **Test MAE /eV** | **Test RMSE /eV** | **Test R²** |
| --- | --- | --- | --- |
| **CRY_ENCODER+MLP** | **0.265** | **0.374** | **0.902** |
| SchNet | 0.421 | 0.629 | 0.763 |
| XGBoost | 0.477 | 0.684 | 0.720 |
| ALIGNN | 0.400 | 0.586 | 0.782 |
| CGCNN | 0.573 | 0.832 | 0.584 |
| RandomForest | 0.627 | 0.872 | 0.543 |
| Mean Baseline | 0.936 | 1.196 | -0.001 |

**Table S4. Ablation studies isolating the contributions of specific architectural components and hyperparameters.** The table details the performance variations when omitting the contrastive pretraining phase, the PAEP encoder, or the Weighted Self-Attention (W-SA) / equivariant modules from the complete StructPot-CLR framework. Additionally, it evaluates the model's sensitivity to varying graph message-passing cutoff radii (from 4 to 10 Å). The results quantitatively validate that the joint structure-PAEP alignment is the primary driver of predictive accuracy, while a cutoff radius of 8 Å optimally encompasses necessary long-range electrostatic interactions without introducing excessive computational overhead.

| **Model Variant** | **WF MAE ↓** | **WF RMSE** | **WF R² ↑** |
| --- | --- | --- | --- |
| w/o Contrastive Pretraining | 0.428 | 0.636 | 0.758 |
| w/o PAEP Encoder | 0.608 | 0.858 | 0.557 |
| w/o W-SA / Equivariant Module | 0.423 | 0.631 | 0.761 |
| Cutoff Radius = 4 Å | 0.384 | 0.569 | 0.796 |
| Cutoff Radius = 6 Å | 0.267 | 0.378 | 0.900 |
| Cutoff Radius = 8 Å | 0.265 | 0.374 | 0.902 |
| Cutoff Radius = 10 Å | **0.264** | **0.374** | **0.902** |
